# Between faith and psychosis: The case of a traditional healer with schizoaffective disorder

**DOI:** 10.1192/j.eurpsy.2026.11459

**Published:** 2026-07-31

**Authors:** G. Chakchouk, N. Regaieg, Y. Trabelsi, F. Guermazi, I. Feki, J. Masmoudi, I. Baati

**Affiliations:** Department of Psychiatry A, Hedi Chaker Hospital, Sfax, Tunisia

## Abstract

**Introduction:**

Cultural frameworks influence how psychiatric symptoms are experienced, expressed, and interpreted both on the personal and community level.

In Tunisian society, belief in the paranormal is endorsed by the strong religious teachings and it’s most prevalent in rural areas.

**Objectives:**

This study highlights the complex overlap between culturally shaped experiences and psychotic manifestations in a patient recognized as a traditional healer within her community.

**Methods:**

We report the case of a patient hospitalized in psychiatry A unit of Hedi Chaker University Hospital, in Sfax (Tunisia).

**Results:**

It was Ms. N.G., a 42-year-old woman, who was admitted for public disturbance following a three month progression of behavioral disorganization and mystical speech. She had a known psychiatric history of schizophrenia with three previous psychiatric hospitalizations.

At admission, she presented with euphoric mood, psychomotor agitation, pressured speech, racing thoughts, and auditory hallucinations. Her discourse revolved around communication with jinn—supernatural entities recognized in Islamic culture—and the conviction that she was divinely chosen to heal others. She described performing healing rituals and diagnosing others as “possessed.”

Social investigation revealed that she was well known locally as a spiritual healer and highly respected for her supposed abilities. Family members and neighbors described her as “closer to God.”

While her community perceived her experiences as a sacred gift, clinicians recognized them as part of a psychotic process. She initially refused medication, fearing it would block her healing powers.

After clinical evaluation, a diagnosis of schizoaffective disorder of bipolar type was established.

She was treated with haloperidol, Sodium valproate, and clonazepam. Over one month, her agitation decreased, insight partially improved, and she resumed organized social behavior. However, her belief in jinn communication and her healing abilities persisted.

The coexistence of socially valued religious experiences and pathological thought distortion created a diagnostic and therapeutic dilemma. Management required cultural sensitivity, collaboration with family, and respect for spiritual meaning while ensuring psychiatric stabilization.

**Conclusions:**

Psychiatric assessment in transcultural settings must integrate sociocultural context into the diagnostic process. Using culturally informed frameworks such as the Cultural Formulation Interview of the DSM-5 (Diagnostic and statistical manual of mental disorders, 5^th^ edition), can help differentiate pathological beliefs from culturally normative experiences. This case demonstrates that recognizing cultural meanings is not an obstacle but a clinical necessity for effective, ethical, and respectful psychiatric care.

**Disclosure of Interest:**

None Declared

